# Metabolic Syndrome and Bone: Pharmacologically Induced Diabetes has Deleterious Effect on Bone in Growing Obese Rats

**DOI:** 10.1007/s00223-017-0367-z

**Published:** 2017-12-01

**Authors:** Cedo M. Bagi, Kristin Edwards, Edwin Berryman

**Affiliations:** 1 Pfizer WRD, Comparative Medicine, Global Science and Technology, 100 Eastern Point Road, Groton, CT 06340 USA; 2 Pfizer R&D, Global Science and Technology, 100 Eastern Point Road, Groton, CT 06340 USA

**Keywords:** Obese diabetic rats, Streptozotocin, Liver fibrosis, Bone structure, Bone strength

## Abstract

Metabolic syndrome and osteoporosis share similar risk factors. Also, patients with diabetes have a higher risk of osteoporosis and fracture. Liver manifestations, such as non-alcoholic steatohepatitis (NASH), of metabolic syndrome are further aggravated in diabetics and often lead to liver failure. Our objective was to create a rat model of human metabolic syndrome and determine the long-term impact of early-onset T1D on bone structure and strength in obese growing rats. Male rats were given either standard chow and RO water (Controls) or a high-fat, high-cholesterol diet and sugar water containing 55% fructose and 45% glucose (HFD). A third group of rats received the HFD diet and a single dose of streptozotocin to induce type 1 diabetes (HFD/Sz). Body weight and glucose tolerance tests were conducted several times during the course of the study. Serum chemistry, liver enzymes, and biomarkers of bone metabolism were evaluated at 10 and 28 weeks. Shear wave elastography and histology were used to assess liver fibrosis. Cancellous bone structure and cortical bone geometry were evaluated by mCT and strength by the 3-point bending method. Body mass and fat accumulation was significantly higher in HFD and HFD/Sz rats compared to Controls. Rats in both the HFD and HFD/Sz groups developed NASH, although the change was more severe in diabetic rats. Although both groups of obese rats had larger bones, their cancellous structure and cortical thickness were reduced, resulting in diminished strength that was further aggravated by diabetes. The HFD and HFD/Sz rats recapitulate MeSy in humans with liver pathology consistent with NASH. Our data provide strong indication that obesity accompanied by type 1 diabetes significantly aggravates comorbidities of MeSy, including the development of osteopenia and weaker bones. The juvenile rat skeleton seems to be more vulnerable to damage imposed by obesity and diabetes and may offer a model to inform the underlying pathology associated with the unusually high fracture rates in obese adults with diabetes.

## Introduction

A growing number of adults worldwide are being diagnosed with metabolic syndrome (MeSy) characterized by a combination of abdominal obesity, dyslipidemia, hyperglycemia, and hypertension [1]. The same conditions are also postulated as risk factors for osteoporosis, another disease with increasing incidence that is a frequent comorbidity in patients with MeSy [2, 3]. The prevalence of type 1 and type 2 diabetes is also growing worldwide, and despite normal to high bone mineral density (BMD) [4], patients with both types of diabetes have increased risk of osteoporosis and fractures [5, 6]. Moreover, treatment of bone fractures in diabetic patients is also more costly due to prolonged healing and other complications [7].

MeSy can be induced in rodents using a high-sucrose/high-fat diet that recapitulates the commonly used diet in Western countries containing high levels of sucrose, lipids, fatty acids, cholesterol, sodium, and chloride [8]. Rodents fed a westernized diet are frequently used as models of obesity, metabolic syndrome, and osteoporosis [9]. Likewise, streptozotocin-induced diabetes in rodents is a well-established animal method recapitulating the key characteristics of type 1 diabetes (T1D) in humans [10]. Streptozotocin has a selective capacity to enter pancreatic β-cells via the glucose transporter GLUT2 and cause DNA damage and deplete nicotinamide adenine phosphate and ATP, ultimately leading to necrosis of β-cells and diabetes [11]. Streptozotocin-induced diabetes negatively impacts bone homeostasis favoring the maturation of bone marrow adipocytes at the expense of osteoblast lineage, thereby causing bone loss driven by impaired bone formation rather than by increased bone resorption [12–14]. In clinics, occurrence of diabetes in patients with MeSy signals the turning point at which the disease takes a more malignant course with worsening of symptoms caused by liver failure [1]. Although animal models of obesity and diabetes are well described, combination of both diseases in a single model is lacking. Development of diabetes in obese patients is an important turning point that usually marks the beginning of more aggressive disease and development of MeSy [1]. Therefore, the objective of this exploratory study was to establish a rat model of MeSy in humans and determine the long-term impact of early-onset T1D and mild hyperglycemia on the structural and mechanical properties of bones in obese growing rats. The results from this study would then be used to design subsequent studies that will better determine consequences of mild and severe diabetes on progression of liver disease and osteoporosis in obese rats as well as to develop strategy for combination therapies. We hypothesized that the combination of T1D and obesity will have a long-lasting deleterious effect on the growing skeleton and will worsen extraskeletal symptoms of MeSy.

## Materials and Methods

### Animals and Study Design

Thirty-six male CD, Sprague Dawley rats were acquired from Charles River Labs (Raleigh, NC) at 4 weeks of age and were acclimated for 1 week prior to being used in the 28-week study. All procedures performed on these animals were in accordance with regulations and established guidelines that were reviewed and approved by the Pfizer Institutional Animal Care and Use Committee. The rats were housed in pairs in isolator cages in a temperature- and humidity-controlled room on a regular 12-h light/dark cycle. The rats were given either standard chow and reverse osmosis (RO) water or a high-fat, trans-fat diet with 2% cholesterol (HFD) (ENVIGO custom diet TD.160178, Madison, WI) and sugar water at 42 g/l (55% fructose and 45% glucose, w/w) as the water source [8]. The HFD diet generates 45% calories from fat (23% fat by weight and 50 IU/kg vitamin E) with 30% of the fat in the form of partially hydrogenated vegetable oil (6.6% trans-fat by weight). Body weight was measured twice weekly throughout the study. Food and water consumption was measured for a 24-h period prior to the scheduled endpoint. The rats were allocated to the study groups based on their body weight. A group of 12 rats received standard chow and RO water (Group 1, Control) and another group of 12 rats received HFD chow and sugar water (Group 2; HFD). The third group of 12 rats also received HFD chow with sugar water and a single intraperitoneal injection of streptozotocin (Sz) (Group 3; HFD/Sz). Animals were fasted 6 h prior to Sz injection (30 mg/kg; Sigma Chemical Company, St. Louis, MO) during week 4 of the study. Immediately prior to injection, Sz was dissolved in 50 mM sodium citrate buffer (pH 4.5) to a final concentration of 30 mg/ml and administered within 5 min, using 1-ml syringes and 23-gauge needles [14]. An equal volume of citrate buffer was intraperitoneally administered to rats in the study groups 1 and 2. Ten days after the administration of Sz, blood glucose was measured using a glucometer (AlphaTRAK, Zoetis Inc., Kalamazoo, MI) in all study animals via tail vein nicking to ensure that the effect of Sz was achieved in all injected rats.

### Glucose Tolerance Test

Rats were fasted overnight for approximately 16 h. Baseline blood samples were collected for determination of insulin and glucose prior to oral administration of 2 g/kg (5 ml/kg) glucose. Glucose levels were measured at 15 and 30 min and at 1, 2, and 3 h post glucose dosing. An additional 50 μl of blood was collected at each time point for the determination of serum insulin level.

### Body Weight, Tissue Collection, and Serum Analyses

The body weight was recorded twice weekly throughout the study. A subgroup of four rats from groups 1–3 was euthanized at the end of 10 weeks, and the remaining eight rats per study group were euthanized at the end of week 28. The purpose of euthanizing a subgroup of rats from each group at 10 weeks was to evaluate the effect of the diet on the development of liver steatohepatosis and to cross-calibrate serum biomarkers of liver function with liver histology and sheer wave elastography and was reported for informational purposes only. At necropsy, the entire right hind limb was harvested and carefully cleaned of all soft tissue. The limbs were wrapped in saline-soaked gauze and frozen at − 20 °C for ex vivo imaging and histological analyses of the cortical and cancellous bone. Blood was collected at the end of the study by jugular venipuncture under isoflurane anesthesia. The serum was stored at − 20 °C and used to run a standard chemistry panel and assess biomarkers of bone metabolism. The serum chemistry analysis included alanine aminotransferase (ALT), glutamic oxaloacetic transaminase (AST), alkaline phosphatase (ALP), cholesterol (CHOL), high-density lipoprotein (HDL), low-density lipoprotein (LDL), triglycerides (TRIG), glucose (GLUC), insulin (INS), albumin (ALB), globulin (GLOB), albumin/globulin ratio (ALB/GLOB), blood urea nitrogen (BUN), creatinine (CREA), phosphorus (P), calcium (Ca), sodium (Na), potassium (K), and chloride (Cl).

Serum osteocalcin was measured using a rat EIA kit (Cat# BT-490, Biomedical Technologies, Stoughton, MA) and procollagen type 1 *N*-terminal propeptide (P1NP) was quantified using the LC/MS method [15]. The level of cross-linked type I collagen C-terminal telopeptide (CTX–I) was analyzed using a rat LAPS^™^ Assay (Cat# AC-06F1).

### Magnetic Resonance Imaging (MRI)

Body composition, fat mass, and lean mass were analyzed via EchoMRI (The EchoMRI^™^ 4in1-500, Houston, TX) prior to scheduled endpoint. Conscious rats were placed in restraining tubes to restrict movement, and then scan was performed; the total scan time was approximately 40–60 s. Fat mass and lean mass were measured from the whole body data by excluding bone, fur, and nails.

### Ultrasound Imaging

Shear wave elastography (SWE) is used to assess liver fibrosis in vivo [16]. In short, the ‘push’ induced in the tissue by acoustic radiation force, which travels sideways through the tissue as a shear wave, is utilized to measure elastic properties or ‘stiffness’ of the liver. SWE imaging was conducted by means of a SuperSonic Imagine—Aixplorer (Aix-en-Provence, France) using a Broadband 4–15 MHz probe, software version 10.1.1.1837, to image the right medial lobe of the liver immediately prior to the scheduled endpoint. SWE measurements were obtained from a region of interest 4.00 mm in diameter, with a mean of five measurements used for statistical analysis. The hepatorenal index was also utilized to assess for signs of hepatosteatosis [17]. An image capturing both the right kidney and the right liver lobe was obtained in B mode. A region of interest 2.0 mm in diameter was selected at the same depth of both the liver and the kidney to calculate the ratio.

### Micro-Computed Tomography (mCT)

The femur and tibia were cleaned off all soft tissues and carefully separated. The tibia was then cut above the tibiofibular junction, and the proximal tibia was placed in a custom-made plastic positioning device to ensure consistent scanning utilizing a MicroCT 100^®^ computed tomography system (Scanco Medical, Bassersdorf, Switzerland). The cancellous bone compartment of the proximal tibial metaphysis was analyzed 1 mm below the growth plate and extended 3 mm distally to include the metaphyseal secondary spongiosa. In short, an ROI was drawn on 100 consecutive slices with a thickness of 1.0 mm that best represented the central segment of the tibia [18]. The cancellous bone parameters included bone mineral density (BMD), tissue volume (TV; bone and bone marrow), bone volume (BV), bone volume/tissue volume ratio (BV/TV), bone surface (BS), trabecular number (TNo), trabecular thickness (TTh), trabecular separation (TSp), connectivity diameter (ConnD), and structural model index (SMI).

### mCT Evaluation of the Cortical Bone

The cortical bone parameters were evaluated at mid-diaphysis (midshaft) with a MicroCT 100^®^ system using a previously described method [18, 19]. Sample measurements (scans) were performed on 25 slices (1 slice = 10.5 μm) using high-resolution settings, and the average value was used for the final calculation. The following parameters were evaluated at the cortical mid-diaphysis: bone mineral density (BMD; g/cm^2^), tissue volume (T. Volume; mm^3^), bone volume (B. Volume; mm^3^), bone marrow volume (BM. Volume; mm^3^), cortical thickness (C. Th; mm), bone area (B. Area; mm^2^), polar moment of inertia (pMoI; mm^4^), maximal I value (I_MAX_; mm^4^), and minimal I value (I_MIN_; mm^4^) and I_MAX_/C_MAX_ (I_MAX_/C_MAX_; mm^3^).

### Bone Strength Testing Using the 3-Point Bending Method

Both the left and right femurs were mechanically tested using the 3-point bending method (Instron materials testing machine (5543A, Instron Inc., Norwood, MA). The femurs were positioned cranial side up across two lower contacts that had a span of 5–7 mm, with an upper contact centered between the lower contacts. The bone was broken by 3-point bending using a cross-head speed of 0.5 mm/min [19]. During testing, force and displacement data were collected at a frequency of 200 Hz using the BlueHill 3 testing software version 3.41 (Instron Inc.). Force/displacement curves were generated, and ultimate force (maximum load, N), stiffness (maximum slope, N/mm), and energy to fracture (N mm) were recorded.

### Histology of Liver and Pancreas

The scarified rats were quickly dissected, and entire livers and pancreas were removed and fixed in 10% neutral formalin for 24 h followed by dehydration in an increasing gradient of alcohol, clearing in xylene, and embedding in paraffin. Sections were cut by a microtome at 5 μm thickness and dried on glass slides. The sections were deparaffinized in xylene and hydrated in water with a descending series of ethyl alcohol. Staining was performed using hematoxylin and eosin (H&E) for examination of the nucleus and cytoplasm. Sirius red, combined with morphometry, was used to quantify collagen using Bioquant image analysis software (Bioquant Image Analysis Software, Nashville, TN). Immunohistochemical staining of alpha smooth muscle actin (αSMA) and desmin was performed on formalin-fixed, paraffin-embedded liver sections with a rabbit polyclonal antibody (Abcam, Cambridge, England), and for the detection of antibody against insulin in the pancreas, an avidin–biotin–peroxidase system was used. Specifically, the primary antibody used was mouse monoclonal anti-insulin antibody (Medico Company, Egypt) at a dilution of 1:100, which was incubated with the slides for 1 h at room temperature followed by counterstaining with Meyer’s hematoxylin. A pathologist blinded to the experimental protocol scored random areas per slide for necrosis, inflammation, and dysplasia.

### Statistical Analysis

Data are expressed as the mean ± SEM. Differences between groups were analyzed with one-way analysis of variance (ANOVA). Statistical differences between the vehicle group and the HFD or HFD/Sz group were evaluated with Dunnett’s multiple comparison test for those parameters that exhibited *P* ≤ 0.05. Statistical comparisons between the HFD and HFD/Sz groups were performed by unpaired *t* test. For all tests, *P* < 0.05 was considered statistically significant. GraphPad Prism version 5.0 for Windows (GraphPad Software, USA, http://www.graphpad.com) was used for the statistical analysis.

## Results

### Body Weight and Body Composition

**Fig. 1:**
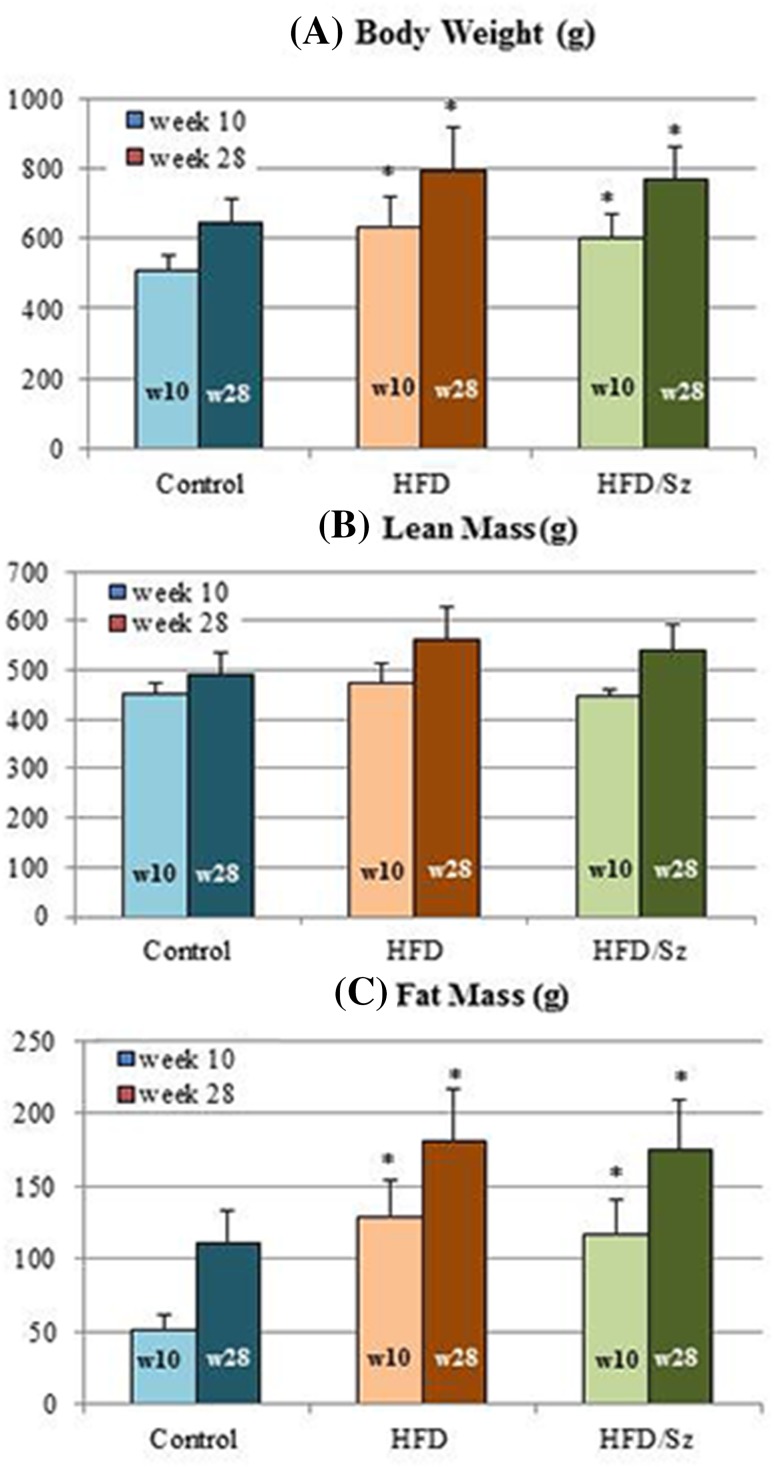
Change in body weight, lean body mass, and fat body mass of Control, HFD, and HFD/Sz rats at week 10 (w10) and week 28 (w28) of the study. Data are presented as mean ± SEM. *P < 0.05 versus Control rats by Dunnett’s multiple comparison test

The body weight of all rats used in the study increased during the course of the study. At the 10-week time point (w10), both HFD and HFD/Sz rats showed higher body weight and higher fat mass relative to the Control animals; however, the lean mass was similar in all rats regardless of diet and treatment with Sz. At week 28 (w28), rats on HFD diet and rats on HFD diet treated with Sz exhibited higher body weight and fat mass relative to the Control animals; however, the lean mass was similar in all rats regardless of treatment (Fig. 1).

### Serum Chemistry

Ten weeks following initiation of the study, both HFD and HFD/Sz rats showed significantly higher levels of cholesterol, triglycerides, and low-density lipoprotein, while they showed lower levels of high-density lipoprotein. In addition, the Sz-treated rats exhibited elevated serum levels of alkaline phosphatase and glucose relative to those of Control and HFD rats (Table 1). There were no differences in serum levels of phosphorus, calcium, sodium, potassium, and chloride (data not shown).

**Table 1:** Selected serum chemistry parameters measured at weeks 10 and 28 of the study. Data are presented as mean ± SD

Parameter	Unit	Control (Group 1; *n* = 4)	HFD (Group 2; *n* = 4)	HFD/Sz (Group 3; *n* = 5)
Week 10				
ALT	U/l	43.8 ± 2.02	39.0 ± 3.82	40.5 ± 2.81
AST	U/l	115.8 ± 13.18	115.0 ± 17.64	110.0 ± 11.34
CHOL	mg/dl	71.8 ± 3.44	142.5 ± 14.63*	133.0 ± 12.86*
TRIG	mg/dl	108.3 ± 19.21	210.0 ± 35.88*	424.0 ± 76.95*^,a^
HDL	mg/dl	51.8 ± 73.23	29.3 ± 2.79*	22.5 ± 1.09*
LDL	mg/dl	6.3 ± 0.22	13.0 ± 2.19*	10.3 ± 1.97*
ALP	U/l	204.3 ± 21.16	240.3 ± 19.74	349.0 ± 26.82*^,b^
GLUC	mg/dl	167.0 ± 14.25	157.5 ± 18.38	208.0 ± 9.34^*,a^
ALB	g/dl	3.7 ± 0.09	4.0 ± 0.13	3.8 ± 0.13
INSU	ng/ml	1.0 ± 0.44	1.2 ± 0.51	1.0 ± 0.39
GLOB	g/dl	2.6 ± 0.08	3.2 ± 0.25	3.1 ± 0.21
ALB/GLOB	Ratio	1.4 ± 0.05	1.3 ± 0.05	1.3 ± 0.05
CREA	mg/dl	0.3 ± 0.00	0.3 ± 0.00	0.3 ± 0.00
BUN	mg/dl	12.75 ± 0.50	13.75 ± 1.50	16.25 ± 3.30
Osteocalcin	ng/ml	253.9 ± 7.54	366.7 ± 20.62*	291.5 ± 18.31*^,a^
P1NP	ng/ml	247.1 ± 13.22	332.3 ± 19.51*	248.8 ± 16.56^a^
CTX-I	ng/ml	21.6 ± 1.89	24.3 ± 1.64	19.1 ± 0.98

Parameter	Unit	Control (Group 1; *n* = 8)	HFD (Group 2; *n* = 8)	HFD/Sz (Group 3; *n* = 8)
Week 28				
ALT	U/l	29.3 ± 2.31	55.6 ± 3.77^*^	70.4 ± 6.28*^,a^
AST	U/l	78.4 ± 7.94	135.5 ± 17.94*	180.1 ± 27.31*^,a^
CHOL	mg/dl	77.8 ± 4.12	135.5 ± 11.36*	180.3 ± 22.19*
TRIG	mg/dl	175.1 ± 23.18	153.6 ± 21.86	146.1 ± 31.08
HDL	mg/dl	57.5 ± 4.73	44.3 ± 7.19	58.9 ± 12.83
LDL	mg/dl	6.00 ± 0.76	12.4 ± 1.89*	16.0 ± 2.11*
ALP	U/l	90.8 ± 3.88	152.4 ± 16.31*	134.1 ± 20.89*
GLUC	mg/dl	183.1 ± 17.25	169.1 ± 14.80	189.6 ± 9.37
INSU	ng/ml	1.5 ± 0.73	1.0 ± 0.75	0.9 ± 0.65
ALB	g/dl	3.5 ± 0.12	3.7 ± 0.30	3.7 ± 0.28
GLOB	g/dl	2.7 ± 0.25	3.1 ± 0.19	3.3 ± 0.28
ALB/GLOB	Ratio	1.3 ± 0.09	1.2 ± 0.08	1.1 ± 0.06
CREA	mg/dl	0.3 ± 0.05	0.3 ± 0.05	0.4 ± 0.07
BUN	mg/dl	16.63 ± 2.00	14.88 ± 1.46	14.38 ± 2.80
Osteocalcin	ng/ml	112.8 ± 7.94	188.7 ± 15.84*	190.1 ± 8.64*
P1NP	ng/ml	85.6 ± 9.13	205.3 ± 13.69*	143.3 ± 7.73*^,a^
CTX-I	ng/ml	12.1 ± 0.17	13.8 ± 1.21	12.4 ± 0.62

* *P* < 0.05 versus Control rats by Dunnett’s multiple comparison test

^a^ *P* < 0.05 versus HFD rats by unpaired *t* test

At the 10-week time point, serum osteocalcin was elevated in both the HFD and HFD/Sz groups of rats relative to Controls, although the highest values were recorded in the HFD group. The markers of bone formation Osteocalcin and P1NP were both elevated in the HFD group relative to both Control and HFD/Sz groups. There were no differences in CTX-I levels among the groups (Table 1).

At week 28, both HFD and HFD/Sz rats showed significantly higher levels of ALT and AST, cholesterol, low-density lipoprotein, and alkaline phosphatase, although the difference was more obvious in the Sz-treated group of HFD rats (Table 1).

At the 28-week time point, serum osteocalcin was elevated in both the HFD and HFD/Sz groups relative to Controls. The marker of bone formation P1NP was elevated in the HFD and HFD/Sz groups relative to the Control group; however, the highest values were recorded in the HFD group. There were no differences in CTX-I levels among the groups (Table 1).

### Liver Imaging and Histology

**Fig. 2:**
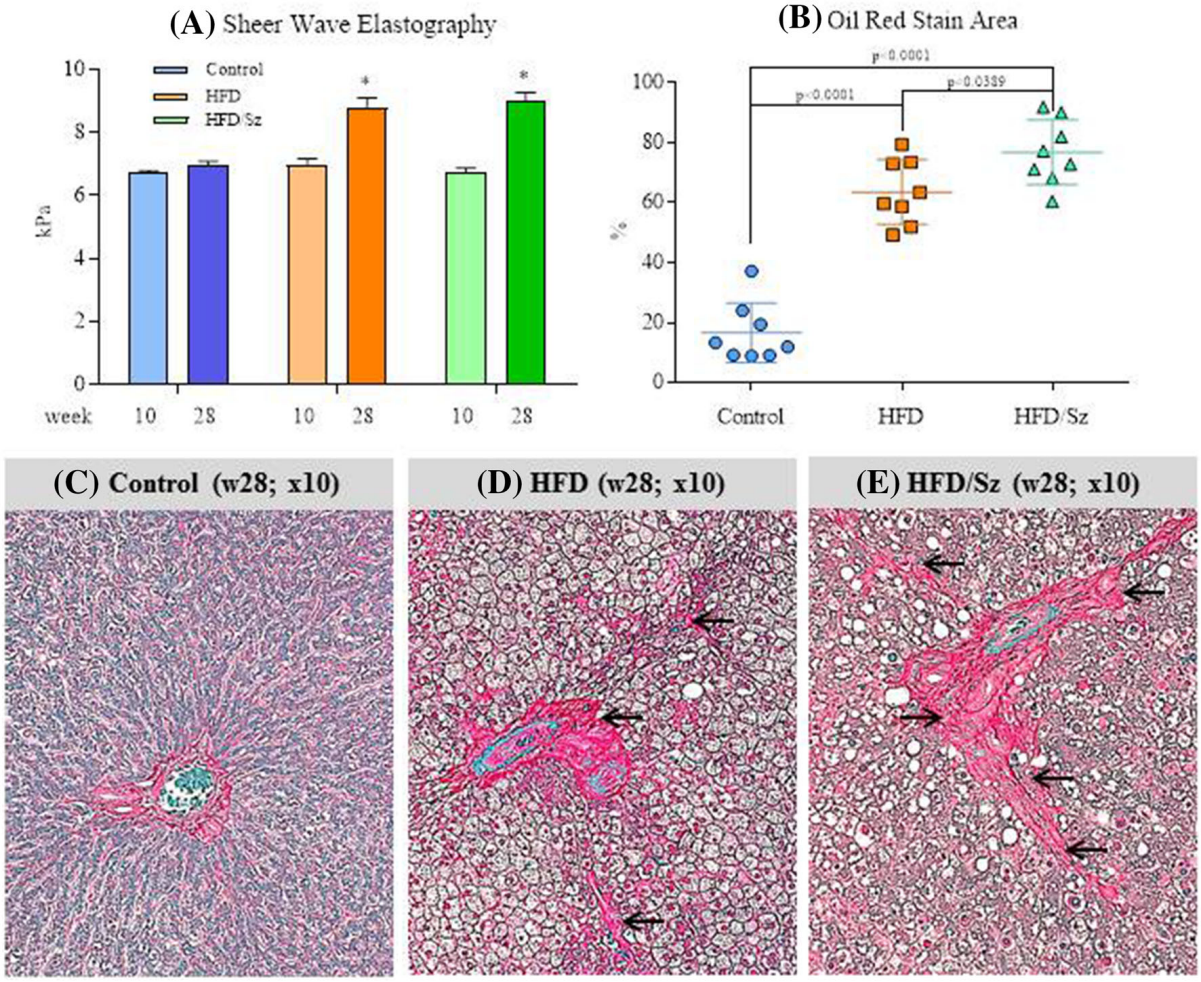
Change in elastic properties of rat livers from week 10 to 28 measured by shear wave elastography. There is a clear increase in resistance of the liver tissue (stiffness) in HFD and HFD/Sz rats compared to Control rats (**c**–**d**). Histological evaluation demonstrates the presence of steatohepatosis and the initiation of perisinusoidal fibrosis with inflammation and occasional ballooning in rats fed HFD diet. More pronounced steatohepatitis with perisinusoidal fibrosis, ballooning, and fibrosis is evident in HFD/Sz rats (**a** Picrosirius red; arrows indicate perisinusoidal fibrosis). In addition, Morphometry of liver sections stained with Oil Red staining shows difference between rats fed a HFD and Control rats, but also more aggressive disease in HFD/Sz rats relative to HFD rats (**b** *P < 0.05 versus Control rats by Dunnett’s multiple comparison test; ^a^ *P* < 0.05 versus HFD rats by unpaired *t* test)

Despite histological evidence of steatosis in the HFD and HFD/Sz rats at week 10 of the study (data not shown), SWE results did not show differences in liver stiffness among the three study groups. At week 28, however, livers from both HFD and HFD/Sz rats showed a significant increase in stiffness relative to the livers of Control rats, and the change in stiffness was more evident in the Sz-treated rats (Fig. 2). Histological findings of the livers at the 28-week time point paralleled the SWE data, as the livers from both HFD and HFD/Sz rats exhibited hallmarks of NASH including macro- and microsteatohepatosis, ballooning, inflammation, and fibrosis. Similar to the SWE data, histology showed more advanced NASH in the HFD/Sz-treated group relative to the HFD group (Fig. 2).

### Histology of the Pancreas and Glucose Tolerance Test

**Fig. 3:**
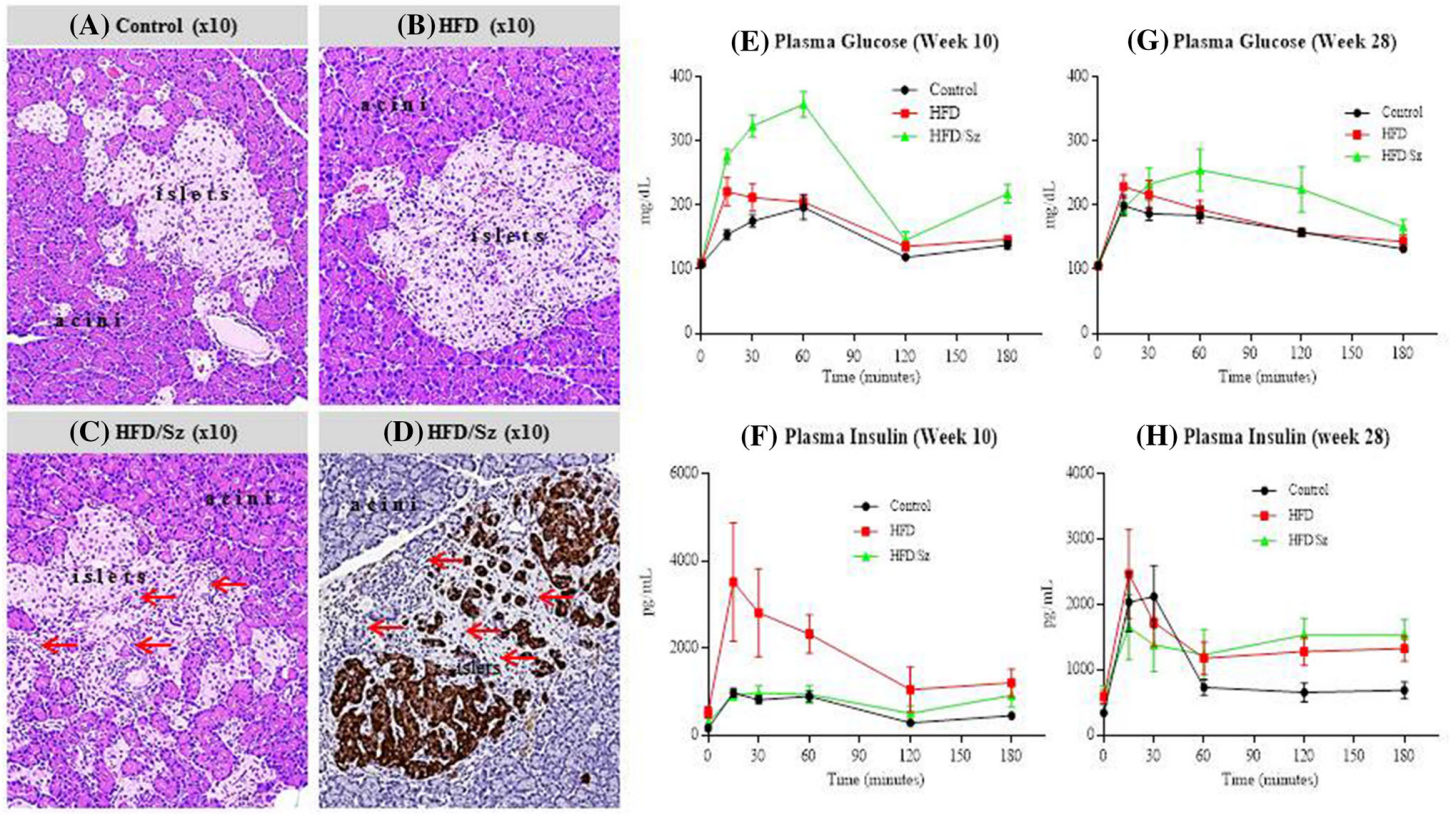
Examples of exocrine (acini) and endocrine (islets of Langerhans) pancreas from Control (**a**), HFD (**b**), and HFD/Sz (**c**) rats at week 10 of the study. Arrows indicate apoptotic β-cells and scar tissue in the pancreas of Sz-treated rats (**c**). Effect of Sz on pancreas is also clearly visible by immunohistochemistry (**d**) with insulin-stained β-cells (dark brown) and occasional presence of scar tissue replacing apoptotic β-cells indicated by red arrows. For determination of insulin and glucose levels, the rats were fasted overnight, bled the next morning to collect baseline serum, and then dosed with water (Controls) or sugar water (HFD and HFD/Sz). Serum was collected at various intervals for determination of insulin and glucose. Sz-treated rats showed clear evidence of type I diabetes mellitus at week 10 (**e** and **f**) but this effect was diminished at week 28 (**g** and **h**)

Sz-treated HFD rats developed type I diabetes mellitus as evidenced by both GTT and histological and histochemical examinations (Fig. 3). Even though the rats in the HFD/Sz group received a single injection of Sz at the very beginning of the study, the insufficiency of β-cells in the islets of Langerhans persisted throughout the study, although the insulin deficiency became less severe over time as the rats grew older, and the β-cells had gradually recovered from injury.

### Analysis of Cancellous Bone

**Fig. 4:**
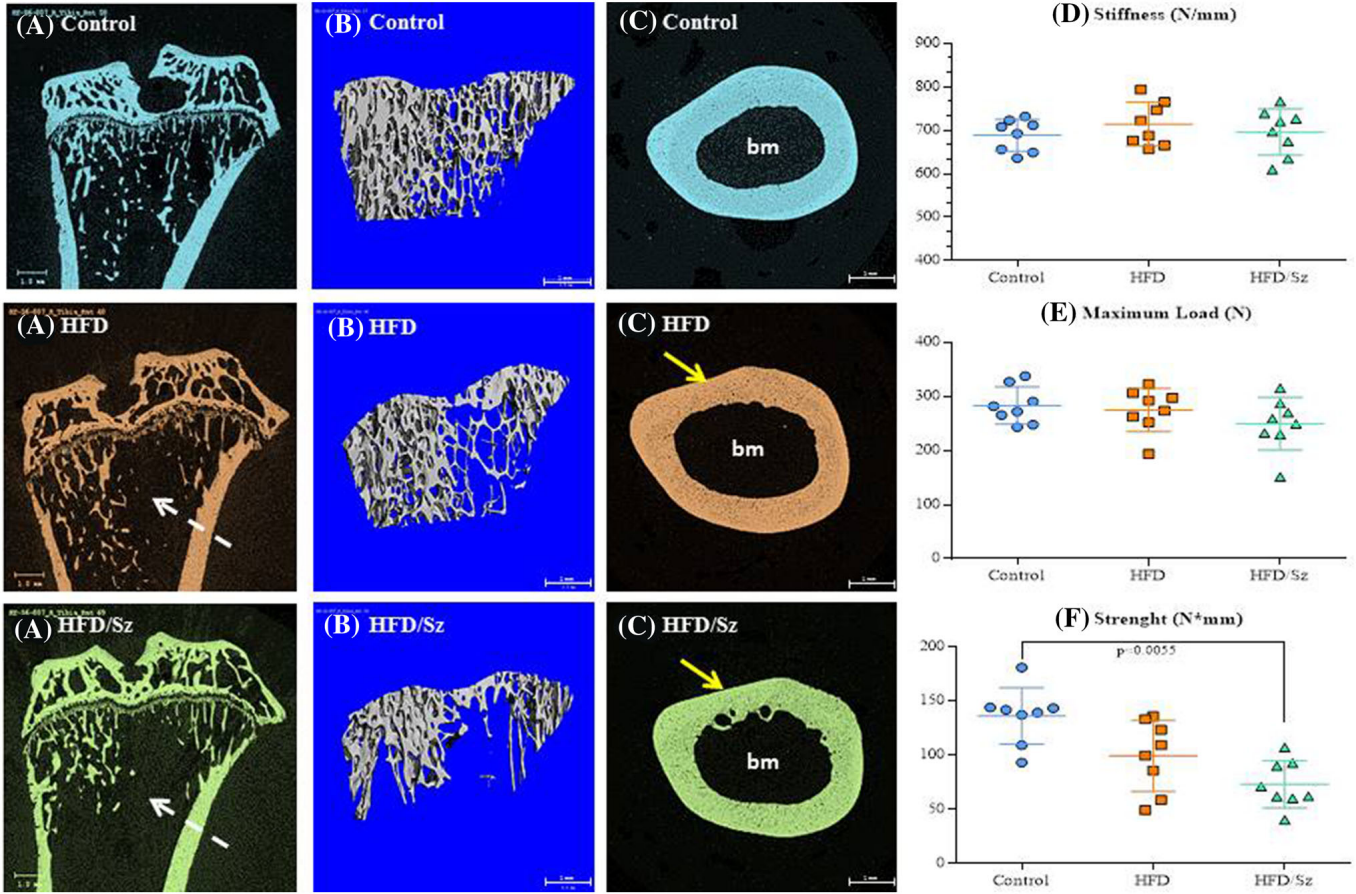
The left column shows 2D mCT images of the proximal tibia at week 28 of the study. The white arrows indicate a reduced amount of cancellous bone in the secondary spongiosa in tibias from rats in the HFD and HFD/Sz groups. The central column depicts 3D images of the cancellous bone from proximal tibial metaphysis in the area of secondary spongiosa showing fewer trabeculae in rats from the HFD and HFD/Sz groups. The right column shows cross-sections of the cortical bone from femoral mid-diaphysis. Rats from both the HFD and HFD/Sz groups show larger bone marrow area (bm) and thinner cortical bone indicated by red arrows compared to Controls. Graphs depicting bone strength of the femoral mid-diaphysis using a 3-point bending method are depicted far right. *P < 0.05 versus Control rats by Dunnett’s multiple comparison test

Even though the rats fed a HFD had slightly longer tibias at 10 weeks, geometry of the long bones was similar between rats in all the study groups. Micro-CT analysis of the cancellous bone from proximal tibial metaphysis (secondary spongiosa) revealed a significant decrease in bone volume, BV/TV ratio, bone surface, trabecular number, and connectivity density index and increased trabecular separation in both HFD and HFD/Sz rats relative to the Control animals and at both time points, weeks 10 and 28. The effect of the westernized diet was more pronounced in rats treated with Sz relative to the untreated HFD animals (Table 2, Fig. 4a, b).

**Table 2:** Cancellous bone parameters obtained by mCT of secondary spongiosa from proximal tibial metaphysis at weeks 10 and 28 of the study. Data are presented as mean ± SEM

Parameter	Unit	Control (Group 1; *n* = 4)	HFD (Group 2; *n* = 4)	HFD/Sz (Group 3; *n* = 5)
Week 10				
Tibial length	mm	40.8 ± 2.8	43.4 ± 3.0	40.2 ± 1.6
Tibial width	mm	4.3 ± 0.4	4.6 ± 0.4	4.2 ± 0.3
BMD	mg/cm^3^	977 ± 9	973 ± 6	994 ± 8
Tissue volume	mm^3^	15.31 ± 0.2	17.05 ± 0.2	17.04 ± 0.4
Bone volume	mm^3^	2.06 ± 0.2	1.12 ± 0.1*	1.19 ± 0.2*
BV/TV	ratio	0.14 ± 0.02	0.07 ± 0.02*	0.07 ± 0.02*
Bone surface	mm^2^	91.06 ± 9.4	52.25 ± 6.5*	58.30 ± 8.5*
Trabecular number	1/mm	2.994 ± 0.4	1.532 ± 0.1*	1.288 ± 0.2*
Trabecular thickness	mm	0.046 ± 0.002	0.043 ± 0.002	0.043 ± 0.003
Trabecular separation	mm	0.312 ± 0.07	0.645 ± 0.06*	0.693 ± 0.3*
SMI	1	2.36 ± 0.13	2.71 ± 0.03*	2.45 ± 0.07
Connectivity density	1/cm^3^	70.49 ± 8.6	22.74 ± 4.2*	22.04 ± 6.9*

Parameter	Unit	Control (Group 1; n = 8)	HFD (Group 2; n = 8)	HFD/Sz (Group 3; n = 8)
Week 28				
Tibial length	mm	44.1 ± 2.2	44.8 ± 2.6	43.8 ± 2.0
Tibial width	mm	4.8 ± 0.2	4.8 ± 0.3	4.6 ± 0.2
BMD	mg/cm^3^	1036 ± 4	1027 ± 7	1035 ± 4
Tissue volume	mm^3^	15.75 ± 0.4	17.33 ± 0.9	16.60 ± 0.8
Bone volume	mm^3^	2.05 ± 0.3	1.28 ± 0.2*	1.16 ± 0.1*
BV/TV	ratio	0.13 ± 0.03	0.07 ± 0.01*	0.07 ± 0.01*
Bone surface	mm^2^	67.86 ± 7.5	43.86 ± 8.4*	41.12 ± 5.9*
Trabecular number	1/mm	2.201 ± 0.08	1.203 ± 0.22*	1.022 ± 0.15*
Trabecular thickness	mm	0.059 ± 0.01	0.060 ± 0.01	0.056 ± 0.01*
Trabecular separation	mm	0.441 ± 0.08	0.855 ± 0.19*	0.931 ± 0.20*
SMI	1	2.18 ± 0.1	2.28 ± 0.1	2.17 ± 0.1
Connectivity density	1/cm^3^	34.06 ± 7.9	14.68 ± 3.2*	11.36 ± 2.8*

* *P* < 0.05 versus Control rats by Dunnett’s multiple comparison test

^a^ *P* < 0.05 versus HFD rats by unpaired *t* test

### Cortical Bone Geometry

Micro-CT analysis of the cortical bone at femoral mid-diaphysis (midshaft) showed decreased cortical thickness and increased endosteal perimeter in HFD and HFD/Sz rats compared to Control rats. Even though periosteal perimeter and polar moment of inertia were increased in HFD and HFD/Sz rats relative to Controls, the change did not reach statistical significance (Table 3). Images of the cortical bone cross-sections confirmed the bone geometry data obtained by mCT and revealed thinner cortex and larger perimeters on both cortical envelopes in HFD and HFD/Sz animals compared to those in Controls (Fig. 4).

**Table 3:** Cortical bone parameters obtained by pQCT at femoral mid-diaphysis at weeks 10 and 28 of the study. Data are presented as mean ± SEM

Parameter	Unit	Control (Group 1; *n* = 4)	HFD (Group 2; *n* = 4)	HFD/Sz (Group 3; *n* = 5)
Week 10				
BMC	mg	12.17 ± 1.1	11.74 ± 0.3	11.33 ± 0.4
BMA	mm^2^	8.81 ± 0.7	8.57 ± 0.11	8.25 ± 0.2
BMD	mg/cm^2^	1379 ± 6	1368 ± 8	1374 ± 8
Thickness	mm	0.85 ± 0.04	0.80 ± 0.02*	0.77 ± 0.01*
Periosteal Pm	mm	13.07 ± 0.05	13.59 ± 0.06	13.41 ± 0.05
Endosteal Pm	mm	7.75 ± 0.08	8.13 ± 0.12*	8.10 ± 0.15*
pM0I	mm^4^	27.05 ± 0.7	28.53 ± 0.9	27.56 ± 1.1
I_MAX_	mm^4^	19.17 ± 1.9	19.87 ± 0.4	19.51 ± 1.8
I_MIN_	mm^4^	11.68 ± 0.8	11.57 ± 0.3	11.67 ± 06

Parameter	Unit	Control (Group 1; *n* = 8)	HFD (Group 2; *n* = 8)	HFD/Sz (Group 3; *n* = 8)
Week 28				
BMC	mg	14.62 ± 0.8	15.07 ± 0.9	14.73 ± 0.6
BMA	mm^2^	10.03 ± 0.5	10.46 ± 06	10.23 ± 0.4
BMD	mg/cm^2^	1456 ± 6	1440 ± 6	1439 ± 6
Thickness	mm	0.95 ± 0.06	0.91 ± 0.03*	0.81 ± 0.02*^,a^
Periosteal Pm	mm	13.35 ± 0.2	14.33 ± 0.3*	13.04 ± 0.4
Endosteal Pm	mm	7.28 ± 0.3	8.44 ± 0.3*	8.29 ± 0.4*
pM0I	mm^4^	37.58 ± 2.2	41.41 ± 2.9	36.54 ± 2.6
I_MAX_	mm^4^	30.53 ± 2.1	32.91 ± 4.8	27.84 ± 2.2
I_MIN_	mm^4^	14.77 ± 0.9	16.10 ± 0.9	15.99 ± 1.3

* *P* < 0.05 versus Control rats by Dunnett’s multiple comparison test

^a^ *P* < 0.05 versus HFD rats by unpaired *t* test

### Cortical Bone Strength

Cortical bone strength measured by the 3-point bending method showed a significant reduction in HFD and HFD/Sz rats relative to Controls. In addition, cortical strength was somewhat lower in the HFD/Sz rats compared to that in the HFD rats. There were no statistically significant differences between the study groups in maximal load and stiffness (Fig. 4).

## Discussion

Hyperglycemia resulting from insulin deficiency or insulin resistance is frequently accompanied with obesity and dyslipidemia in patients with metabolic syndrome [1]. Occurrence of hyperglycemia further worsens comorbidities associated with abdominal obesity including non-alcoholic fatty liver disease (NAFLD) and NASH. Both diseases can be simulated in an animal model so that the negative effect of hyperglycemia on bone metabolism in obese patients can be adequately studied. Although the nature of NAFLD and NASH in rodents depends on the kind of diet used to induce the disease, the general pathology of the key organs replicates many aspects of metabolic syndrome in humans [9, 20]. In our study, rats fed an HFD diet showed signs of NAFLD by week 10, and afterward the disease progressed further so that by the end week 28, all HFD-fed rats showed elevated liver enzymes and more fibrotic and stiffer liver with histological evidence of steatohepatosis and perisinusoidal fibrosis. Similar to patients with metabolic syndrome with concomitant diabetes, induction of hyperglycemia in obese rats further impaired liver metabolism and accelerated progression of liver fibrosis toward the NASH phenotype.

Overall, healthy obesity is not necessarily detrimental for the skeleton, as increased mechanical loading on weight-bearing bones usually improves BMD, making the bones less susceptible to fractures [21, 22]. However, aggressive expansion of adipose tissue in the liver and other organs leads to uncontrolled secretion of adipokines that can negatively impact overall metabolism and inflammatory responses throughout the body [23, 24]. For example, the shift of differentiation of mesenchymal stem cells toward adipocytes and accumulation of adipocytes in the bone marrow negatively impact bone metabolism [25–27]. Prolonged feeding of rats with the HFD diet in our study negatively affected both cancellous and cortical bone, as demonstrated by the loss of trabecular bone number and impaired geometry of the cortical bone. The reduced strength of the cortical bone in obese rats was not surprising, as it is well established that thinning of the cortical bone reduces bone strength and typically results in increased propensity to fracture [19, 28]. Bones that are bigger are usually stronger and more rigid (stiffer) due to periosteal apposition that adds bone farther from the neutral axis [28, 29], but in our case, despite the increase in size and polar moment of inertia, strength of the cortical bones from HFD and HFD/Sz rats was greatly reduced relative to bones from rats fed a regular chaw. The results of imaging and bone strength analyses from this study are in accordance with previously published data that clearly demonstrated diminished formation and increased resorption in obese rodents resulting in impaired bone architecture and diminished bone strength [30–33].

A growing body of clinical and preclinical studies cites diabetes as an independent indicator of higher risk of fracture [5–8, 12–14]. We showed that the induction of mild T1D in obese rats intensifies the loss of bone architecture and reduces the ability of cortical bone to adequately compensate for increased weight bearing, leading to weaker bones. Delayed bone accrual, reduced bone size and cortical thickness, elevated porosity of the cortex, and altered bone matrix deposition are frequently cited as the most plausible reasons for weaker bones in diabetic rats [34, 35]. Similar to rodents, the higher propensity to fractures in diabetic patients could also result from higher than normal cortical porosity, thinner cortex, or change in material properties of the bone tissue [36, 37]. Mechanisms proposed to drive bone pathology in diabetic patients include deficiency in the secretion of insulin and insulin-like growth factor 1, impaired regulation of vitamin D and parathyroid hormone, and emergence of hyperglycemia [38, 39]. Collectively, preclinical and clinical data suggest that diminished proliferation and differentiation of bone marrow mesenchymal stem cells into osteoblasts, suppressed osteoblast function, and increased non-enzymatic glycation of collagen can further compromise bone architecture and bone strength in obese diabetics.

Factors contributing to obesity, as well as obesity itself, seem to be more detrimental to adolescents during intensive skeletal development based on clinical data showing that excessive fat accumulation in obese children often leads to compromised skeletal development [40]. Clinical data also show that patients with T1D frequently develop mild osteopenia and lower BMD and have disproportionately higher risk of fracture as adults [41]. The growth plates at the epiphyses of long bones in rodents remain open throughout their lifespan resembling characteristics of juvenile human skeleton. Accordingly, bone metabolism in rodents and human adolescents is characterized by modeling-driven bone formation rather than by bone remodeling, which is a hallmark of bone metabolism in adults [19]. The results from this study demonstrate that growing obese rats, similarly to obese children, quickly develop less favorable bone architecture with reduced strength [42] and that the presence of T1D diminishes the ability of growing skeletons to properly adapt its structure and geometry to meet mechanical demands imposed by increased weight, ultimately leading to weaker bones in adults.

Since the mCT results clearly revealed loss of the trabeculae in the bone compartment and cortical bones at the endosteal envelope, we postulate that the increased osteocalcin and P1NP in rats fed an HFD diet results from intense bone modeling at the cortical periosteum directed to accommodate the increase in weight rather than an increase in bone remodeling. In that regard, the lower levels of bone formation markers at week 28 seem logical, since the rats were becoming older, and their growth had reached physiologic limits.

Fibril-forming type I and III collagen are the most abundant proteins found in the extracellular matrix (ECM) of the liver [43]. During uncontrolled fibrogenesis, type I collagen levels increase up to eightfold, and the ratio of the type I/III collagen changes from 1:1 in the healthy liver to 1:2 in the fibrotic liver [44]. Myofibroblasts play a crucial role in the wound healing process, and they are the main producers of collagen in chronic liver injury such as NAFLD and NASH. The main sources of myofibroblasts are hepatic stellate cells, but an additional source of myofibroblasts includes bone marrow-derived monocytes and fibrocytes [45]. Based on high serum P1NP and the very nature of the NAFLD model used in this study, it cannot be excluded that the serum P1NP could, at least partially, reflect the extraskeletal synthesis of type I collagen and therefore P1NP could potentially be utilized as a surrogate biomarker of liver fibrosis.

Taken together, our data further confirmed the negative relationship between obesity, bone marrow adipogenesis, diabetes, and the development of NASH. Results from this study strongly indicate that T1D accompanied with obesity significantly aggravates comorbidities of MeSy, including the development of osteopenia and weaker bones. The juvenile skeleton seems to be more vulnerable to damage imposed by obesity and diabetes, which could lead to unusually high fracture rates in obese adults with diabetes. In that regard, rodent models of NASH are valuable preclinical tools to study comorbidities associated with MeSy in humans. Finally, additional studies are warranted to elucidate the mechanism by which comorbidities such as obesity and diabetes affect the liver and skeleton, but also other organ systems. In that regard, studies aimed to validate the complex models of MeSy should be of appropriate duration (months rather than weeks) to allow for the disease to take place in order to adequately study disease pathophysiology and test novel therapies.
